# The Relation of Cytotoxin-Associated Gene-A Seropositivity with Vitamin B12 Deficiency in *Helicobacter pylori*-Positive Patients

**DOI:** 10.1155/2019/1450536

**Published:** 2019-12-09

**Authors:** Celal Ulasoglu, Hatice Esin Temiz, Zuhal Aydan Sağlam

**Affiliations:** ^1^Department of Gastroenterology, Istanbul Medeniyet University Goztepe Training and Research Hospital, Istanbul, Turkey; ^2^Department of Family Medicine, Istanbul Medeniyet University Goztepe Training and Research Hospital, Istanbul, Turkey

## Abstract

**Background and Aim:**

As a worldwide infectious bacterium, *H. pylori* leads to stomach pathologies such as gastritis, peptic ulcer, gastric cancer, MALToma, and various extragastric manifestations. In our study, we aimed to investigate the association between serum vitamin B12 level and cytotoxin-associated gene-A (CagA) seropositivity, which is one of the virulence factors of *Helicobacter pylori* (*H. pylori*).

**Method:**

This study has been conducted on 289 patients who have met the inclusion criteria. Within these patients, 213 of them were *H. pylori* positive and 76 were negative. Vitamin B12 and CagA-IgG levels were assessed in consecutive dyspeptic patients undergoing upper endoscopy.

**Results:**

Out of 289 patients, 51.9% were women (*n* = 150) and *H. pylori* was detected in 213 (73.7%) patients. Histopathological evaluation with modified Sydney classification revealed lymphocyte infiltration in 66.8% (*n* = 193), activation in 46% (*n* = 133), metaplasia in 11.4% (*n* = 33), atrophy in 11.4% (*n* = 33), and lymphoid follicles in 21.1% (*n* = 61) of the patients. Within *H. pylori*-positive patients, the ratio of CagA positivity was 57.3% (*n* = 122). Low B12 vitamin level was significantly correlated with existence of *H. pylori* (*p*=0.02), CagA (*p*=0.002), lymphocyte (*p*=0.006), metaplasia (*p*=0.001), atrophy (*p*=0.001), and lymphoid follicles (*p*=0.006). Positivity of CagA has been detected to be statistically corelated with lymphocyte (*p*=0.001) and activation (*p*=0.005); however, the same relation was not present with atrophy (*p*=0.236).

**Conclusion:**

In conclusion, B12 deficiency was positively correlated with CagA positivity and gastric inflammatory activity.

## 1. Introduction and Aim

*Helicobacter pylori* (*H. pylori*) is present in almost 50% of the world's population [1], while in western world it is up to 20% and about 90% in developing countries [2, 3]. It is a Gram (−), unipolar, spiral or curved, mobile, rounded tip, with 4–6 flagella, microaerophilic, and sausage-like bacteria. It contains urease enzyme, which allows it to colonize in the mucus layer [4, 5]. In addition, many studies suggest that *H. pylori* can cause extragastric diseases such as iron deficiency anemia [6], immune thrombocytopenic purpura [7], ischemic heart disease [8], diabetes mellitus, insulin resistance [9], Parkinson's disease [10], Alzheimer's disease [11], acne rosacea [12], and vitamin B12 deficiency [13–15]. In our study, we aimed to investigate the association between serum B12 vitamin level (tested in patients as a part of dyspepsia investigation) and presence of virulence factor cytotoxin-associated gene-A (CagA) seropositivity to clarify the relation with the type of histological damage whether it is atrophy or inflammation. Also, we aimed to assess the histological reflection of vitamin B12 deficiency.

## 2. Materials and Methods

The patients investigated with upper gastrointestinal endoscopy due to dyspepsia were analysed for their data of demographic, clinical, endoscopic, and histopathological findings and levels of vitamin B12 and CagA IgG. Exclusion criteria were pregnancy, malabsorption syndromes, chronic liver disease, chronic renal failure, organ failure, being vegetarian, history of gastric surgery, inflammatory bowel disease (IBD), regular use of proton pump inhibitor (PPI), and patients receiving vitamin supplementation. A total of 76 *H. pylori*-negative and 213 *H. pylori*-positive patients gave written and informed consent and agreed to participate in the study. Blood from the antecubital vein was collected from patients in the morning after 8–12 hours of fasting. Blood samples were centrifuged for 10 min at 2000 g and stored at −80 centigrade until study. The vitamin B12 and folate levels in serum were assessed by chemiluminescent immunoassay, and levels below 150 pg/mL and 3 ng/ml, respectively, were regarded as deficient. In serum samples, CagA IgG analysis was performed using ELISA method using commercial kit (Dia-Pro-Sel Italy) according to the manufacturer's instructions. The interassay CV of the assay was in the range of 4–20%. False positivity rate was less than 2%. Endoscopic biopsy materials from corpus and antrum were fixed with 10% formaldehyde and were evaluated with hematoxylin and eosin stain and Giemsa. *H. pylori* presence was determined histologically. The statistical evaluation was done by NCSS (Number Cruncher Statistical System, Kaysville, Utah, USA, 2007). Student's *t*-test was used for the comparison of descriptive statistical methods (mean, standard deviation, median, frequency, and ratio) as well as between groups; Mann–Whitney *U* test was used for the evaluation of those who did not have normal distribution. Pearson's chi-square test, Yates Continuity Correction, and Fisher's exact test were used to compare qualitative data. Multivariate binary logistic regression model is used to assess the prediction of independent variables on dependent categorical values. ROC Curve Analysis was used to evaluate CAG titer according to pathological findings. The results were evaluated at 95% confidence interval and *p* < 0.05 at significance level. All authors had access to the study data and reviewed and approved the final manuscript.

## 3. Results

The study was conducted on a total of 289 patients: 51.9% (*n* = 150) female and 48.1% (*n* = 139) male patients. The mean age was 44.8 ± 15.8 (18–75) years.

Smokers were 21.8% (*n* = 63) of the subjects. The mean vitamin B12 level was 292.7 ± 143.8 pg/ml (50–804). The mean MCV measurement of the subjects was 85.7 ± 7.1 (31.6–117.0) femtoliter (fL). The mean folate level was 8.9 ± 3.6 ng/ml (1.9–19.5). Histopathological examination revealed *H. pylori* positivity in 73.7% (*n* = 213), lymphocyte in 66.8% (*n* = 193), activation in 46% (*n* = 133), metaplasia in 11.4% (*n* = 133), atrophy in 11.4% (*n* = 33), and lymphoid follicle in 21.1% (*n* = 61). There was no difference of vitamin B12 between genders (Table 1). CagA antibody was negative in 42.7% (*n* = 91) and positive in 57.3% (*n* = 122) of the cases. The mean CagA antibody titer was 27.12 ± 35.29 (0.1–154.7) arb/ml. Of CagA-positive patients, 18.8% (*n* = 40) had low titer while 38.5% (*n* = 82) had high titer. According to the presence of *H. pylori*, no statistically significant difference was found between the mean age, gender difference, MCV values, smoking, or BMI of the patients (*p* > 0.05) (Table 1). The folate and B12 levels were found to be significantly lower in *H. pylori*-positive patients (*p*=0.003), while CagA-positive cases had low vitamin B12 levels compared to CagA-negative cases, but no difference was assessed in folate levels (*p*=0.001) (Tables 2 and 3).

The higher ratio of lymphocyte infiltration (*p*=0.008), inflammatory activity (*p*=0.005), and significantly low B12 levels (*p*=0.006) were found in patients with CagA positivity (*p*=0.001 and *p*=0.006, respectively) (Tables 4 and 5). There was no statistically significant difference between the atrophy rates and CagA positivity (*p*=0.320) (Table 5). The mean B12 levels of the CagA-positive patients with lymphocytes (*p*=0.006), activation (*p*=0.012), metaplasia (*p*=0.109), atrophy (*p*=0.001), and lymphoid follicle (*p*=0.033) were lower than those of the CagA-negative cases (Table 4). MCV and folate levels were not related to B12 value (*r* = −0.06, *p*=0.30 and 0.110, *p*=0.06, respectively).

CagA titer measurement was significantly higher in patients with lymphocytes and activation (*p*=0.001, *p*=0.01). There was no statistically significant difference between the CagA titer measurements and presence of metaplasia, lymphoid follicle, and atrophy (*p* > 0.05). A statistically significant difference was found between B 12 measurements in terms of CagA antibody status in female patients (*p*=0.017, *p* < 0.05). The B12 values were lower in male patients with CagA-positive than CagA-negative male cases (*p*=0.001). Binary logistic regression model revealed that activation was significantly more predictive in CagA(+) patients than the presence of atrophy for B12 deficiency (*p*=0.037 versus *p*=0.708, respectively).

## 4. Discussion

*H. pylori* leading to gastritis, peptic ulcer, gastric cancer, gastric lymphoma, iron deficiency anemia, pernicious anemia, autoimmune thrombocytopenia, and growth retardation is a common infection [6, 7, 9, 10]. The negative effect of *H. pylori* on serum vitamin B12 levels is reported in a number of studies in adults. Reduction of acid-pepsin secretion due to *H. pylori*-induced atrophic gastritis [16], bacterial overgrowth in the intestine [17], reduction of intrinsic factor [18], and antibody development against canalicular and parietal cells [19, 20] are some of the proposed mechanisms. To our knowledge, the relationship between CagA and vitamin B12 interaction was not reported previously. In this study, we aimed to investigate the relationship between vitamin B12 levels and *H. pylori* virulence factor CagA.

In the study, intestinal metaplasia, presence of lymphocytes, glandular atrophy, activation, and presence of lymphoid follicles were significantly higher in *H. pylori*-positive group compared to *H. pylori*-negative group (*p* < 0.05). Many studies have found a relationship between the presence of *H. pylori* and glandular atrophy in the gastric mucosa [21].

In our study, B12 vitamin levels were found to be significantly lower in *H. pylori*-positive cases (*p* < 0.05). Gulsen et al. found that *H. pylori* seroprevalence was 77% and 68.6% in low and normal vitamin B12 levels, respectively, but not statistically significant (*p*=0.06). The researchers attributed this result of serological parameters without active infection [22, 23]. In our study, confirmation of active infection by endoscopy eliminates this confusion. Captain et al. reported *H. pylori* eradication treated vitamin B12 deficiency in 40% of patients. Although the treatment for vitamin B12 levels is not applied, the improvement of *H. pylori* eradication alone supports the cause and effect relationship and this improvement is reported even in patients without gastric atrophy [15].

In our study, gastric atrophy was found in 11.4% of all cases, who all were *H. pylori* positive. B12 level was significantly lower in cases with gastric atrophy in *H. pylori*-positive patients than those without (*p*=0.001) (Table 4). Reduction of acid-pepsin secretion in *H. pylori*-induced atrophic gastritis decreases the release of vitamin B12 from food-derived proteins (16). In the case of bacterial overgrowth in the hypochlorhydric stomach and intestine, bacteria also utilize vitamin B12. In addition, *H. pylori* increases gastric autoimmunity by causing anticoagulant and antiparietal antibody in the atrophic stomach [19, 20].

In our study, vitamin B12 levels of CagA-positive patients were significantly lower than those of CagA-negative cases (*p* < 0.001). The levels of vitamin B12 were low in the presence of CagA in both female and male subgroups. In addition, in the group with CagA positive, lymphocyte infiltration and inflammatory activation in gastric biopsy were significantly increased. The same relationship could not be demonstrated between atrophy, lymphoid follicles, and metaplasia (Table 5). In our study, the atrophy of CagA is thought to be there with active and chronic inflammation rather than its functional mechanism. Similar to our findings, the recent history of CagA has been linked to gastric inflammation rather than metaplasia or atrophy [24]. CagA causes increase of many cytokines, including IL-8 [25]. Seroprevalence of CagA IgG was 57.3% in patients with *H. pylori* positive.

In our study, no relationship was found between smoking, BMI, age, and sex with *H. pylori* and CagA-IgG positivity (*p* > 0.05). In a study by Afsharipour et al., *H. pylori* and age and sex were not related, but in men and under 20 years of age, they found that CagA-IgG positivity was higher. They suggested that CagA-positive strains were replaced by CagA-negative strains as the age progress may explain the higher incidence of duodenal ulcer and gastric cancer in males [26].

We investigated the level of anti-CagA IgG in 3 groups: patients without CagA, patients with low CagA titer (between 0.1 and 29.9 arbU/mL), and high titer positive cases (>30 arbU/mL). The distribution of the groups was as follows: 91 (42.7%), 40 (18.7%), and 82 (38.5%). In our study, we could not find a relationship between lymphocyte, lymphoid follicle, activation, metaplasia, and atrophy with titer of CagA. This suggests that CagA titer does not alter with the presence of gastric inflammation.

In our study, there was a correlation between the presence of *H. pylori* and B12 level (*p* < 0.05), but not with smoking, sex, age, or BMI (*p* > 0.05). Our findings confirm that vitamin B12 may be associated with gastric inflammation and atrophy. In CagA(+) patients, histological activity was significantly related to B12 deficiency compared to atrophy.

Our study has some limitations to the outcome. Although the patients were asked about their vegetarian eating habits, the individuals in different socioeconomic levels may have different amounts of vitamin B12 with nutrient [27]. This may have affected B12 vitamin levels among our patients. Another limitation was the lack of measuring the active form of vitamin B12, holotranscobalamin (holoTC), which influences DNA synthesizing cells and is comparable with vitamin B12, methyl malonic acid, and total homocysteine levels [28, 29].

In conclusion, we observed that B12 deficiency was associated with both *H. pylori* presence and glandular atrophy in stomach and presence of CagA IgG. To our knowledge and in PubMed query, no study was conducted handling the interaction of B12 and CagA positivity. In our study, CagA IgG is associated with inflammation rather than atrophy, suggesting that CagA causes B12 deficiency with atrophy-independent mechanisms.

## Figures and Tables

**Table 1:** *H. pylori*, CagA, and vitamin B12 status of patients.

	Females, *n* (%)	Males, *n* (%)	Total	*p* value^x^
*n*	150 (51.9%)	139 (48.1%)	289 (100.0%)	*p*=0.47^*∗*^
Age	43.4 ± 15.6	46.5 ± 15.9	44.8 ± 15.8	*p*=0.96^*∗∗*^
*H. pylori* (+)	117 (78.0%)	96 (69.1%)	213 (73.7%)	*p*=0.08^*∗*^
CagA (+) (arbU/mL)	72 (48.0%)	50 (36.0%)	122 (42.2%)	*p*=0.16^*∗*^
Vitamin B12 (pg/ml)	292 ± 139^*∗∗*^	288 ± 139^*∗∗*^	292 ± 143 (50–804)	0.539^*∗∗*^

*H. pylori*: *Helicobacter pylori*; CagA: cytotoxin-associated gene-A. ^+^Females versus males. ^*∗*^Pearson's chi-square test. ^*∗∗*^Student's *t*-test.

**Table 2:** *Helicobacter pylori* status and MCV, folate, and B12 levels.

	*H. pylori*-positive (*n* = 213), mean ± SD	*H. pylori*-negative (*n* = 76), mean ± SD	*p* value
MCV (fl)	86.1 ± 6.0	84.9 ± 7.9	^a^0.188
Folate (ng/dl)	8.3 ± 3.2	10.9 ± 4.0	^a^0.001^*∗∗*^
B 12 (median) (min–max)	276.5 ± 134.0 (256.0)	338.4 ± 160.8 (299.5)	^d^0.003^*∗*^

^a^Student's *t*-test. ^d^Mann–Whitney *U* test. ^*∗*^*p* < 0.05. ^*∗∗*^*p* > 0.01. Min: minimum; Max: maximum; SD: standard deviation; B12: pg/ml; MCV: mean corpuscular volume.

**Table 3:** CagA status and MCV, folate, and B12 levels in *H. pylori* (+) patients.

	CagA-negative (*n* = 91), mean ± SD	CagA-positive (*n* = 122), mean ± SD	*p*
MCV (fl)	86.4 ± 6.1	85.9 ± 7.4	^a^0.631
Folate (ng/ml)	7.9 ± 2.80	8.6 ± 3.5	^a^0.109
B12 (pg/ml)	318.1 ± 149.1	245.6 ± 112.5	^a^0.001^*∗∗*^

^a^Student's *t*-test. ^*∗∗*^*p* < 0.01. SD: standard deviation; MCV: mean corpuscular volume.

**Table 4:** B12 levels in *H. pylori* (+) patients with and without CagA positivity.

	*n* (%)	B12 level (mean ± SD) in patients with CagA (+) *n* = 122	B12 level (mean ± SD) in patients with CagA (−) *n* = 91	*p* value
Total	213 (100%)	245.5 ± 112.5	301.6 ± 146.1	^a^0.003^*∗∗*^
Lymphocyte (+)	181 (85.0%)	243.9 ± 114.8	299.8 ± 154.1	^a^ **0.006** ^*∗∗*^
Activation (+)	133 (62.4%)	244.2 ± 114.6	304.0 ± 153.4	^a^ **0.012** ^*∗∗*^
Metaplasia (+)	33 (15.5%)	222.9 ± 109.4	273.3 ± 135.0	^a^ **0.109** ^*∗*^
Atrophy (+)	33 (15.5%)	168.4 ± 60.5	285.6 ± 134.3	^a^ **0.001** ^*∗∗*^
Lymphoid follicle(+)	61 (28.6%)	234.9 ± 135.5	284.9 ± 127.0	^a^0.033^*∗∗*^

^a^Student's *t-*test. B12*:* pg/ml. ^*∗*^*p* > 0.05. ^*∗∗*^*p* < 0.05. SD: standard deviation.

**Table 5:** CagA serology and relation with histopathological features.

	CagA neg. (*n* = 91)	CagA pos. (*n* = 122)	*p*
	*n* (%)	*n* (%)	
Lymphocyte			^c^ **0.008** ^*∗∗*^
(−)	21 (23.1)	11 (9.0)	
(+)	70 (76.9)	111 (91.0)	

Activation			^b^ **0.005** ^*∗∗*^
(−)	44 (48.4)	36 (29.5)	
(+)	47 (51.6)	86 (70.5)	

Metaplasia			^c^ **0.193**
(−)	73 (80.2)	107 (87.7)	
(+)	18 (19.8)	15 (12.3)	

Atrophy			^c^ **0.320**
(−)	80 (87.9)	100 (82.0)	
(+)	11 (12.1)	22 (18.0)	

Lymphoid follicle			^c^ **0.632**
(−)	67 (73.6)	85 (69.7)	
(+)	24 (26.4)	37 (30.3)	

^b^Pearson's chi-square test. ^c^Yates Continuity Correction. ^*∗∗*^*p* < 0.01.

## Data Availability

The relevant data used to support the findings of this study are available from the corresponding author upon request.

## Abbreviations

CagA: Cytotoxin-associated gene-A
*H. pylori*: *Helicobacter pylori*.

## References

[1] Tsay F.-W., Hsu P.-I. (2018). H. pylori infection and extra-gastroduodenal diseases. *Journal of Biomedical Science*.

[2] Siao D., Somsouk M. (2014). *Helicobacter pylori*: evidence-based review with a focus on immigrant populations. *Journal of General Internal Medicine*.

[3] Hosseini E., Poursina F., de Wiele T. V., Safaei H. G., Adibi P. A. (2012). *Helicobacter pylori* in Iran: a systematic review on the association of genotypes and gastroduodenal diseases. *Journal of Research in Medical Sciences*.

[4] Baskerville A., Newell D. G. (1988). Naturally occurring chronic gastritis and C pylori infection in the rhesus monkey: a potential model for gastritis in man. *Gut*.

[5] Salehi Z., Jelodar M. H., Rassa M. (2008). *Helicobacter pylori* cagA status and peptic ulcer disease in Iran. *Digestive Diseases and Sciences*.

[6] Boyanova L. (2011). Role of *Helicobacter pylori* virulence factors for iron acquisition from gastric epithelial cells of the host and impact on bacterial colonization. *Future Microbiology*.

[7] Stasi R., Sarpatwari A., Segal J. B. (2009). Effects of eradication of *Helicobacter pylori* infection in patients with immune thrombocytopenic purpura: a systematic review. *Blood*.

[8] Tamer G. S., Tengiz I., Ercan E., Duman C., Alioglu E., Turk U. O. (2009). *Helicobacter pylori* seropositivity in patients with acute coronary syndromes. *Digestive Diseases and Sciences*.

[9] Gunji T., Matsuhashi N., Sato H. (2008). *Helicobacter pylori* Infection is significantly associated with metabolic syndrome in the Japanese population. *The American Journal of Gastroenterology*.

[10] Altschuler E. (1996). Gastric *Helicobacter pylori* infection as a cause of idiopathic Parkinson disease and non-arteric anterior optic ischemic neuropathy. *Medical Hypotheses*.

[11] Kountouras J., Boziki M., Gavalas E. (2009). Increased cerebrospinal fluid Helicobacter pylori antibody in alzheimer’s disease. *International Journal of Neuroscience*.

[12] Gürer M. A., Erel A., Erbaş D., Çağlar K., Atahan Ç. (2002). The seroprevalence of *Helicobacter pylori* and nitric oxide in acne rosacea. *International Journal of Dermatology*.

[13] Dholakia K., Dharmarajan T. S., Yadav D. (2005). Vitamin B12 deficiency and gastric histopathology in older patients. *World Journal of Gastroenterology*.

[14] Gümürdülü Y., Serin E., Ozer B. (2003). Predictors of vitamin B12 deficiency: age and *Helicobacter pylori* load of antral mucosa. *Turkish Journal of Gastroenterology*.

[15] Kaptan K., Beyan C., Ural A. U. (2000). *Helicobacter pylori*-is it a novel causative agent in vitamin B12 deficiency?. *Archives of Internal Medicine*.

[16] King C. E., Leibach J., Toskes P. P. (1979). Clinically significant vitamin B12 deficiency secondary to malabsorption of protein-bound vitamin B12. *Digestive Diseases and Sciences*.

[17] Suter P. M., Golner B. B., Goldin B. R., Morrow F. D., Russell R. M. (1991). Reversal of protein-bound vitamin B12 malabsorption with antibiotics in atrophic gastritis. *Gastroenterology*.

[18] Shao J.-S., Sartor R. B., Dial E., Lichtenberger L. M., Schepp W., Alpers D. H. (2000). Expression of intrinsic factor in rat and murine gastric mucosal cell lineages is modified by inflammation. *The American Journal of Pathology*.

[19] Ma J.-Y., Borch K., Sjöstrand S. E., Janzon L., Mårdh S. (1994). Positive correlation between H,K-adenosine triphosphatase autoantibodies and *Helicobacter pylori* antibodies in patients with pernicious anemia. *Scandinavian Journal of Gastroenterology*.

[20] Parente F., Negrini R., Imbesi V. (2001). Presence of gastric autoantibodies impairs gastric secretory function in patients with *Helicobacter pylori*-positive duodenal ulcer. *Scandinavian Journal of Gastroenterology*.

[21] Oksanen A., Sipponen P., Karttunen R. (2000). Atrophic gastritis and *Helicobacter pylori* infection in outpatients referred for gastroscopy. *Gut*.

[22] Gulsen M., Battal A., Uygurer C. (1988). Helicobacter pylori ve kobalamin noksanlığı. *Turkish Journal of Gastroenterology*.

[23] Miehlke S., Yu J., Schuppler M. (2001). *Helicobacter pylori* vacA, iceA, and cagA status and pattern of gastritis in patients with malignant and benign gastroduodenal disease. *The American Journal of Gastroenterology*.

[24] Shiota S., Murakami K., Okimoto T., Kodama M., Yamaoka Y. (2014). Serum *Helicobacter pylori* CagA antibody titer was a useful marker for advanced inflammation in the stomach in Japan. *Journal of Gastroenterology and Hepatology*.

[25] Yamaoka Y., Kita M., Kodama T., Sawai N., Kashima K., Imanishi J. (1997). Induction of various cytokines and development of severe mucosal inflammation by cagA gene positive *Helicobacter pylori* strains. *Gut*.

[26] Afsharipour S., Nazari R., Douraghi M. (2014). Seroprevalence of anti-*Helicobacter pylori* and anti-cytotoxin-associated gene A antibodies among healthy individuals in center of Iran. *Iranian Journal of Basic Medical Sciences*.

[27] Herzlich B., Herbert V. (1988). Depletion of serum holotranscobalamin II. An early sign of negative vitamin B12 balance. *Laboratory Investigation; A Journal of Technical Methods and Pathology*.

[28] Hvas A.-M., Nexo E. (2005). Holotranscobalamin—a first choice assay for diagnosing early vitamin B12 deficiency?. *Journal of Internal Medicine*.

[29] Muhsen K., Sinnreich R., Beer-Davidson G. (2018). Sero-prevalence of *Helicobacter pylori* CagA immunoglobulin G antibody, serum pepsinogens and haemoglobin levels in adults. *Scientific Reports*.

